# P-1575. Trends in antibiotic resistance in community-acquired urinary tract infections among children - a retrospective descriptive nation-wide study

**DOI:** 10.1093/ofid/ofae631.1742

**Published:** 2025-01-29

**Authors:** Michal Stein Yeshurun, Michal Stein, Shirley Shapiro Ben David

**Affiliations:** Sheba Medical Center, Tel Aviv, HaMerkaz, Israel; Sheba Medical Center, Tel Aviv, HaMerkaz, Israel; Maccabi Healthcare Services, Tel Aviv, Tel Aviv, Israel

## Abstract

**Background:**

Urinary tract infection (UTI) is one of the most common bacterial infections in children. Early antibiotic treatment may prevent complications. Empirical treatment requires up-to-date knowledge of the local epidemiology. This study aims to describe trends in resistance rates in community-acquired UTI`s between 2017-2022, assess whether current recommendations for empirical treatment are valid and identify risk factors for resistance.

**Methods:**

Data was collected retrospectively from the computerized system of Maccabi Health Services for all children under 18 years-old diagnosed with a UTI in 2017 and 2022 and analyzed using chi-square test and regression models.

**Results:**

A total of 240,122 urinary samples were included in the study. The most common isolated pathogen was Escherichia coli (78%); there was a slight but significant increase in extended-spectrum beta-lactamases (ESBL) incidence for all Gram-negative Enterobacteriaceae from 6% in 2017 to 8% in 2022, (P-value 0.001). Higher incidence (12%) was detected among *Klebsiella* isolates compared to E. coli (8%). Aminoglycosides resistance rates increased from 5% to 6% (P-value 0.001). The most impressive increase was in resistance to Fluoroquinolones from 7% to 23% (P-value 0.001).

Female gender, younger age, previous antibiotic treatment and urinary tract abnormalities were associated with an increased resistance rate.

**Conclusion:**

The increased resistance rate observed for most pathogens and antibiotics was statistically significant, nevertheless, due to small absolute changes, the recommendations for empirical antibiotic treatment in Israel are still valid. Fluoroquinolones, which are not recommended as empirical treatment, should be avoided due to high levels of resistance.

**Disclosures:**

**Shirley Shapiro Ben David, MD**, GSK: Lecturer|Pfizer: Grant/Research Support|Pfizer: Lecturer

